# Morphological changes of the caudal cervical intervertebral foramina due to flexion-extension and compression-traction movements in the canine cervical vertebral column

**DOI:** 10.1186/s12917-015-0508-4

**Published:** 2015-08-06

**Authors:** Renato M. Ramos, Ronaldo C. da Costa, Andre LA Oliveira, Manoj K. Kodigudla, Vijay K. Goel

**Affiliations:** Department of Veterinary Clinical Sciences, College of Veterinary Medicine, The Ohio State University, 601 Vernon L. Tharp St, Columbus, OH 43210 USA; Department of Clinical and Surgery, Universidade Estadual do Norte Fluminense Darcy Ribeiro, Av. Alberto Lamego, 2000, Campos dos Goytacazes, RJ 28013-602 Brazil; Engineering Center for Orthopedic Research Excellence, The University of Toledo Health Science Campus, 3000 Arlington Ave, Toledo, OH USA

**Keywords:** Cervical spondylomyelopathy, Disc degeneration, Dog, Spinal cord, Wobbler syndrome

## Abstract

**Background:**

Previous studies in humans have reported that the dimensions of the intervertebral foramina change significantly with movement of the spine. Cervical spondylomyelopathy (CSM) in dogs is characterized by dynamic and static compressions of the neural components, leading to variable degrees of neurologic deficits and neck pain. Studies suggest that intervertebral foraminal stenosis has implications in the pathogenesis of CSM. The dimensions of the cervical intervertebral foramina may significantly change during neck movements. This could have implication in the pathogenesis of CSM and other diseases associated with radiculopathy such as intervertebral disc disease. The purpose of this study was to quantify the morphological changes in the intervertebral foramina of dogs during flexion, extension, traction, and compression of the canine cervical vertebral column. All vertebral columns were examined with magnetic resonance imaging prior to biomechanic testing. Eight normal vertebral columns were placed in Group 1 and eight vertebral columns with intervertebral disc degeneration or/and protrusion were assigned to Group 2. Molds of the left and right intervertebral foramina from C4-5, C5-6 and C6-7 were taken during all positions and loading modes. Molds were frozen and vertical (height) and horizontal (width) dimensions of the foramina were measured. Comparisons were made between neutral to flexion and extension, flexion to extension, and traction to compression in neutral position.

**Results:**

Extension decreased all the foraminal dimensions significantly, whereas flexion increased all the foraminal dimensions significantly. Compression decreased all the foraminal dimensions significantly, and traction increased the foraminal height, but did not significantly change the foraminal width. No differences in measurements were seen between groups.

**Conclusions:**

Our results show movement-related changes in the dimensions of the intervertebral foramina, with significant foraminal narrowing in extension and compression.

## Background

Cervical spondylomyelopathy (CSM) is characterized by dynamic and static compressions of the neural components caused by developmental abnormalities and secondary degenerative changes of the cervical vertebral column, leading to variable degrees of neurologic deficits and neck pain [1–5]. The pathogenesis of CSM is not well understood, but it is believed to be multifactorial [6, 7]. Among the factors involved, it has been suggested that intervertebral foraminal stenosis is implicated in the pathogenesis of CSM [5, 8, 9]. It is also known that normal and CSM-affected Dobermans and Great Danes have foraminal stenosis, however, the number of sites and severity is higher in the CSM-affected dogs [5, 9]. It is nonetheless interesting to note that a high proportion of normal dogs have foraminal stenosis without clinical signs. The exact role of foraminal stenosis in the pathogenesis of CSM is still open to debate [5, 9].

Cervical spondylomyelopathy in dogs bears considerable similarities to, and has been proposed as the natural study model for the most common cause of chronic spinal cord dysfunction in humans, called cervical spondylotic myelopathy [4, 10]. In humans, an *in vivo* study with healthy volunteers, measured the dimension of the cervical intervertebral foramina at various positions, showing that flexion significantly increased the foraminal dimensions and extension significantly decreased the foraminal dimensions [11]. These results may explain the clinical observation that cervical extension aggravates symptoms in patients with cervical radiculopathy and that flexion often relieves them [11].

Although the diagnosis and treatment of CSM have been described in the literature, few studies have aimed at understanding the pathogenesis of this disease [5, 8, 12–14]. Experimental studies in dogs have shown that ischemia of the radicular arteries cause severe spinal cord degeneration and necrotic changes [15]. The combination of spinal cord ischemia with compression leads to more severe neurologic deficits than spinal cord compression or ischemia alone [16, 17]. In humans with cervical spondylotic myelopathy, foraminal stenosis is well known to be one of the main causes of cervical pain and ischemic spinal cord injury [18, 19]. It is possible that dynamic foraminal stenosis contributes to the pathogenesis of ischemic injury to the nerve roots and spinal cord in dogs. Extension movements in the cervical vertebral column of dogs causes severe vertebral canal stenosis [20]. Currently, there is no data in the veterinary literature that demonstrates the movement-associated morphological changes of the cervical intervertebral foramina.

The dimensions of the cervical intervertebral foramina may change during neck movements and could be implied in the pathogenesis of diseases associated with cervical radiculopathy such as CSM and intervertebral disc disease. The hypothesis of this investigation was that the dimensions of the intervertebral foramina would increase with flexion and decrease with extension. The objective of this study was to determine the changes in cervical intervertebral foraminal width and height as a result of traction-compression forces and flexion, extension movements in cervical vertebral column specimens of cadaveric dogs. Additionally, we aimed to compare the foraminal changes between normal vertebral columns with vertebral columns with intervertebral disc degeneration or protrusion.

## Results

There were no significant differences in the foraminal height and width between different intervertebral levels, and there were no significant differences between groups 1 and 2 either (group 1 was composed of 8 normal cervical vertebral columns based on the lack of MRI changes, whereas group 2 consisted of 8 vertebral columns with intervertebral disc degeneration or/and disc protrusion at one or more levels in the caudal cervical spine). Since location and groups had no significant influence on the measured intervertebral levels and groups, we will report the results combining the data from both groups and the three intervertebral levels.

### Comparison of neutral vs neutral with compression, and neutral vs neutral with traction

**Fig. 1:**
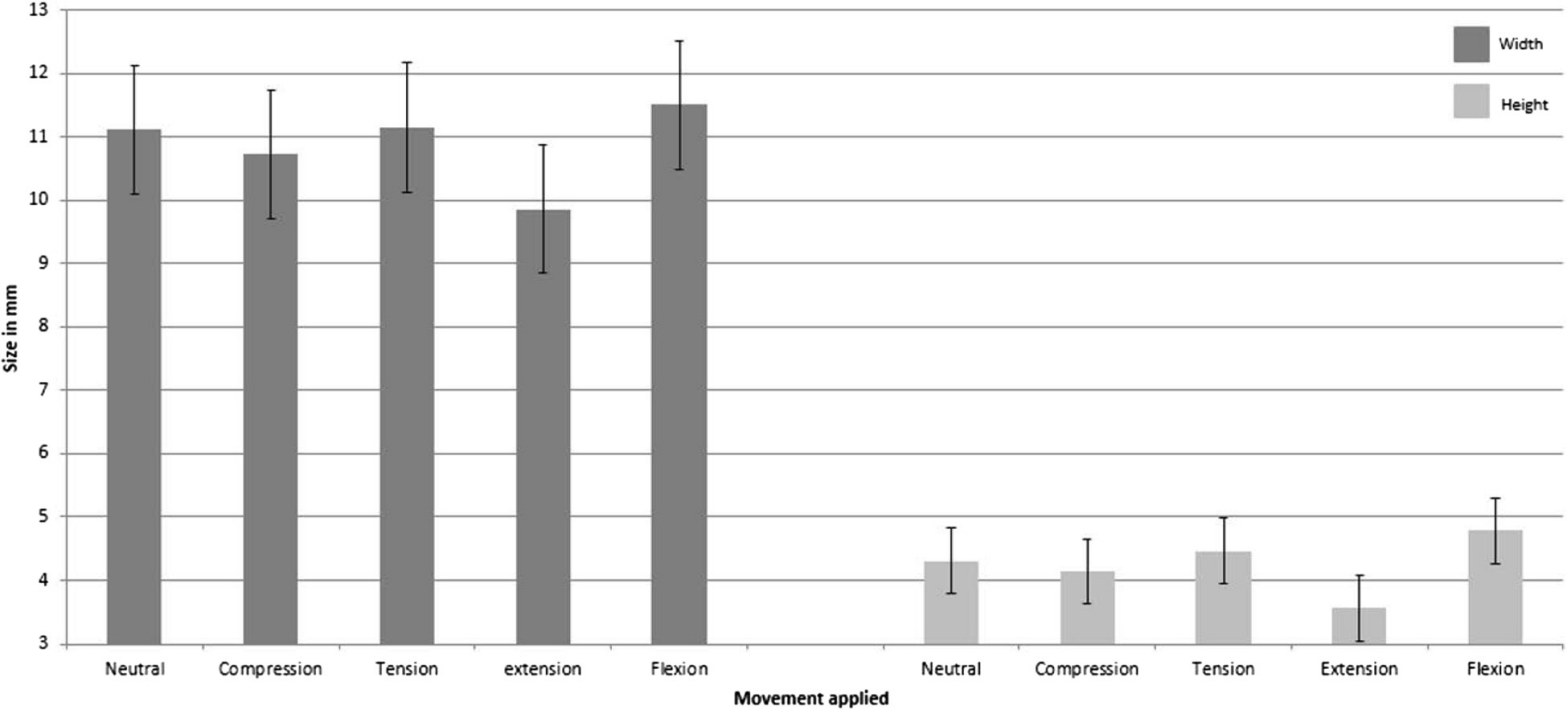
Graph representation of the foraminal width and height changes in neutral, compression, traction, flexion and extension movements. Data represents combined data from all 3 levels (C4-5, C5-6, and C6-7) and all dogs from both groups. Data shown as bar graphs with standard error in millimeters (mm)

In neutral position under no load mean width was 11.12 mm (Table 1). Under compression, the mean width decreased to 10.72 mm (3.6 %.), (*P =* 0.008). With traction, the mean width increased to 11.15 mm, however, this was not significantly different (*P* > 0.99). Moving from neutral position with traction to compression, resulted in a net change of 0.43 mm, (3.9 %) (Fig. 1).

**Table 1:** Mean foraminal width (mm) of the cervical foramina (C4-5, C5-6, and C6-C7), of dogs divided according to movement and load

Movement	Load	Width (mm^a^)	95 % Confidence Interval^a^ (CI)
Neutral	None	11.12	10.61 − 11.62
Neutral	Compression	10.72	10.21 − 11.23
Neutral	Traction	11.15	10.64 − 11.66
Extension	None	9.86	9.35 − 10.36
Flexion	None	11.50	11.00 − 12.01

^a^Foraminal width and CI in millimeters (mm)

In neutral position under no load, the mean height was 4.31 mm (Table 2). Under compression, the mean height decreased to 4.14 mm (4 %), (*P* = 0.004) (Table 3). With traction, the mean height increased to 4.46 mm (3.5 %), (*P* = 0.011). Moving from neutral position with traction to compression resulted in a net change of 0.32 mm (7.4 %).

**Table 2:** Mean foraminal height (mm) of the cervical foramina (C4-5, C5-6, and C6-C7), of dogs divided according to movement and load

Movement	Load	Height (mm)^a^	95 % Confidence. Interval^a^ (CI)
Neutral	None	4.31	4.05 − 4.57
Neutral	Compression	4.14	3.89 − 4.4
Neutral	Traction	4.46	4.20 − 4.72
Extension	None	3.57	3.31 − 3.83
Flexion	None	4.79	4.53 − 5.05

^a^Foraminal height and CI in millimeters (mm)

**Table 3:** Comparison of the results of measurement of height and width of the cervical foramina (C4-5, C5-6, and C6-C7)

Comparisons	Difference^a^ (mm)	95 % Confidence. Interval^a^ (CI)	*p*-value^b^
Height: Neutral/Compression vs. Neutral/None	−0.17	−0.26 − −0.07	0.004
Height: Neutral/Traction vs. Neutral/None	0.15	0.05 − 0.24	0.011
Height: Extension/None vs. Neutral/None	−0.74	−0.84 − −0.64	<0.001
Height: Flexion/None vs. Neutral/None	0.48	0.38 − 0.58	<0.001
Height: Flexion/None vs. Extension/None	1.22	1.12 − 1.31	<0.001
Width: Neutral/Compression vs. Neutral/None	−0.40	−0.64 − −0.16	0.008
Width: Neutral/Traction vs. Neutral/None	0.03	−0.21 − 0.27	>0.99
Width : Extension/None vs. Neutral/None	−1.26	−1.5 − −1.02	<0.001
Width: Flexion/None vs. Neutral/None	0.39	0.15 − 0.63	0.001
Width: Flexion/None vs. Extension/None	1.65	1.41 − 1.89	<0.001

^a^Difference and CI in millimeters (mm)

^b^ *p*-values adjusted by the Holm’s procedure to conserve the overall type I error at 0.05

### Comparison of neutral vs flexion, and neutral vs extension

In neutral position with no load, mean width was 11.12 mm (Table 1). In an extended position, the mean width decreased to 9.86 mm (11.3 %), (*P* < 0.001) (Table 3), whereas in flexion the mean width increased to 11.5 mm (3.5 %), (*P* = 0.001) (Fig. 1).

In neutral position with no load, mean height was 4.31 mm (Table 2). In an extended position, the mean height decreased to 3.57 mm (17.2 %), (*P* < 0.001), whereas in flexion the mean height increased to 4.79 mm (11.1 %), (*P* < 0.001) (Table 3).

### Comparison of flexion vs extension

With regards to comparison between flexion and extension, the mean width changed 1.65 mm (14.8 %), (*P* < 0.001). In terms of comparison between flexion and extension, the mean height changed 1.22 mm (28.3 %), (*P* < 0.001) (Table 3).

### Intraobserver agreement

The intraobserver agreement (rho) for the height and width ratios was 0.86 and 0.97, respectively. These results indicate fair to good agreement for the measurement of all dimensions.

## Discussion

The primary goal of our study was to examine the morphological changes in the intervertebral foramina during flexion, extension, traction, and compression of the cervical vertebral column (C4-C7), and to compare these changes between normal vertebral columns and those with intervertebral disc degeneration. As no differences were seen between groups, the data for all dogs was combined, thus the changes observed seem to affect equally dogs with or without degenerative changes. Disc degeneration and dorsal disc protrusion *per se* does not seem to affect the foramen size, although they could alter the biomechanics of the spine.

In flexion, the cervical foramina width increased 3.5 % and the height increased 11.1 %, when compared with neutral position. In extension, the foraminal width decreased 11.3 % and the height decreased 17.2 % when compared with neutral position. With regards to comparison between flexion and extension, the foraminal width decreased 14.8 %, and the height decreased 28.3 %. These results likely have implications on the pathogenesis of cervical myelopathies because the intervertebral foramina changes were significant in flexion and extension.

Studies *in vitro* and *in vivo* with human cervical vertebral column have shown that flexion significantly increases the width and height dimension of the foramina by 8-16 % and foraminal area by 28 %, whereas extension significantly decreases foraminal dimensions by width and height 10-22 % and foraminal area by 17 % [11, 21]. In general, our results are in agreement with the aforementioned studies. We found a reduction in the foraminal size ranging from 14.8 % to 28.3 % (width and height) going from flexion to extension, respectively.

Although dynamic intervertebral foraminal stenosis has been discussed in recent papers investigating the dynamic component of CSM in dogs, [7, 13] we are the first to document these dynamic intervertebral foraminal changes. Our study shows unequivocally that dynamic movements of the canine cervical vertebral column cause intervertebral foraminal narrowing in extension. This could cause repetitive compression of the radicular arteries and nerves roots, primarily in dogs with intervertebral foraminal stenosis. This dynamic narrowing may be important in the pathogenesis of cervical myelopathies, such as canine CSM, primarily because of the high incidence of foraminal stenosis in CSM-affected dogs [5, 9].

The blood supply to the spinal cord is provided by the radicular arteries that enter the vertebral canal via the intervertebral foramen [15–17]. Intervertebral foraminal stenosis can cause ischemic changes in the spinal cord of dogs. Experimental studies in dogs have shown that the bilateral interruption of the 5^th^ pair of cervical radicular arteries produced spinal cord degeneration in all cervical segments [15]. Other studies have also shown that the effects of spinal cord compression and spinal cord ischemia are additive and cause more neurologic deficits than spinal cord compression or ischemia alone [15–17].

Studies in humans have shown that the local pressure of the intervertebral foramen is significantly higher during extension, of the lumbar vertebral column [22]. Mechanical pressure has the potential to disturb the blood supply and nutrition of the nerve root such that a pressure as low as 10 mmHg was sufficient to decrease the blood supply to the nerve roots, in experimental studies with pigs [23–27]. Others studies suggest that the symptoms of lumbar spinal stenosis, including pain and neurologic deficits, may be the result of ischemia caused by mechanical stress to the spinal nerve root [28–30]. A recent report described 13 cases of cervical intervertebral foraminal disc extrusions. The most common location was C5-6 and C6-7 (70 % cases) and signs were cervical hyperesthesia and lameness. Interestingly, even though the acute disc extrusion caused acute severe foraminal stenosis, most dogs recovered with medical treatment, without surgical decompression of the foramen [31].

A study in humans correlated nerve root compression and decreased foraminal width and area in the cervical vertebral column [32]. This study showed a significant correlation between disc degeneration and reduced foraminal width and foraminal area, but not with foraminal height [32]. In other studies of the lumbar vertebral column, the results showed that narrowing of the disc space significantly reduces the height of the intervertebral foramen but has no significant effects on its width [33]. It is important to remember that humans are bipeds, and foraminal height is equivalent to the width of the foramen in dogs, and the foraminal width in humans equals the height in dogs. We probably did not detect differences in the spines with and without degenerative changes because the number and severity of the degenerative changes in the dogs of group 2 was not sufficiently different from group 1. The incidence of nerve root compression in lumbar vertebral columns was 21 % in when patients had their columns in neutral position, 15.4 % in flexion, and 33.3 % in the extension [34]. In dogs, lumbar foraminal areas were significantly smaller when the vertebral column was extended versus flexed [35, 36].

In humans, degenerative changes of the anatomic structures forming the walls of the intervertebral foramen may affect its dimension and shape causing narrowing or stenosis of the foramen [33, 37]. In a study with 16 normal and 16 CSM-affected Dobermans, foraminal stenosis was detected in 11 clinically normal and 14 CSM-affected dogs [5]. A similar study of 15 clinically normal and 15 CSM-affected Great Danes (CSM-GDs), found foraminal stenosis present in 11 normal and 15 CSM-GDs [9]. Although many normal Dobermans and Great Danes had foraminal stenosis, the number of sites and severity was significantly greater in CSM affected dogs [5, 9]. In our study, there was no significant difference between groups. It is possible that we did not detect differences between groups because even though group 2 had degenerative changes, they probably were less severe than those seen with CSM cases. Most dogs also only had degenerative changes at a single site, whereas dogs with CSM frequently have multiple levels affected.

The application of traction forces is used to treat dogs with vertebral canal stenosis [38, 39]. The goal of these techniques is decompression, by increasing vertebral canal and intervertebral foramen dimensions, by means of traction and stabilization [38, 39]. In the present study, traction forces increased the foraminal height in 3.5 %, which was significant, but the foraminal changes in width were not significant. This finding could partially explain why these techniques lead to clinical improvement in dogs with CSM.

Molds from vertebral column, vertebral canal, and foramina have been used in morphological and biomechanics studies in humans and dogs [40–42]. Oil based clay (EZ Shape, Polyform Products Company) was adopted in this study because it is malleable at room temperature, and becomes rigid when it is frozen. This method was used in a recent study [41]. Vertebral canal and foraminal dimension have been measured by CT, MRI, computer software and caliper [11, 42–46]. In our study, a digital precision caliper was used to measure the foraminal dimensions. This technique has been previously reported to be reliable, with low interobserver and intraobserver variability [33, 45]. A single investigator performed all measurements in a non-blinded manner, which could have biased the observations.

Further studies with larger sample sizes, primarily of dogs confirmed to have CSM with MRI are needed to examine these dynamic changes in affected dogs, and to clarify differences in characteristics and amounts of dynamic change in neural foraminal dimensions between patients with and without cervical radiculopathy.

## Conclusion

Our findings suggest that extension and compressive forces decrease the foraminal height and width. Flexion and traction forces increase the foraminal dimensions. Dynamic foraminal stenosis may play a role in the pathogenesis of cervical myelopathies such as CSM by causing intermittent compression of the radicular arteries, resulting in decreased blood supply to the nerve roots and spinal cord and signs of cervical myelopathy and radiculopathy.

## Methods

### Cadaveric specimen preparation

The investigation was conducted in accordance with the guidelines of the Institutional Animal Care and Use Committee of The Ohio State University. Sixteen intact cervical vertebral columns (C3-T1) were collected from large breed dogs euthanized for reasons unrelated to this study. The specimens were collected from mature canine cadavers from a local shelter. The breeds included in this study were Boxer, Bull Mastiff, Doberman, German Shepard, Labrador, Pit bull, Rottweiler. Body mass averaged 28 kg (25–31 kg) and sex distribution included 12 males and 4 females. All vertebral columns had right lateral fluoroscopic images, followed by magnetic resonance imaging (MRI) with a 3.0 Tesla magnet (Achieva, Philips Healthcare) in neutral position to evaluate vertebral morphology and intervertebral disc hydration. Disc degeneration was established by the visualization of partial or complete loss of the bright signal of nucleus pulposus on sagittal MR images. Intervertebral disc protrusion was defined by dorsal protrusion of the annulus fibrosus causing spinal cord compression. Specimens were divided into 2 groups (8 specimens/group). Normal vertebral columns were placed in Group 1. Group 2 consisted of 8 vertebral columns with intervertebral disc degeneration or/and dorsal disc protrusion at one or more levels in the caudal cervical spine (C4-C7) (Table 4). Specimens were then stripped of all musculature and spinal cord, leaving the vertebral column ligaments and articular facet joint capsules intact. Specimens were wrapped in saline (0.9 % NaCl) solution-soaked towels and stored at -20 °C during two weeks, then thawed for 12 h to room temperature before testing. The specimens were sprayed regularly with sterile physiologic saline solution during mounting and biomechanical testing to prevent desiccation.

**Table 4:** Magnetic resonance changes identified on the 8 vertebral columns of group 2 dogs (dogs with degenerative vertebral changes)

Column	Intervertebral level C4–C5	Intervertebral level C5 – C6	Intervertebral level C6 – C7
1	-	IV^a^ disc degeneration	IV disc degeneration and dorsal disc protrusion
2	IV dorsal disc protrusion	IV disc degeneration and dorsal disc protrusion	-
3	-	-	IV disc degeneration and dorsal disc protrusion
4	-	-	IV disc degeneration and dorsal disc protrusion
5	-	-	IV disc degeneration and dorsal disc protrusion
6	-	-	IV disc degeneration and dorsal disc protrusion
7	-	-	IV disc degeneration and dorsal disc protrusion
8	-	IV disc degeneration	-

^a^IV = intervertebral

For biomechanical testing, screws were inserted into the caudal aspect of the vertebral bodies of T1 for additional support. The caudal end (T1) was potted using bondo, a two part epoxy resin (Bondo, Bondo Corp., Atlanta, GA). This fixed the T1 vertebra of each specimen. A 4 mm hole was drilled into the cranial aspect, across the vertebral body and lamina, of the third cervical vertebra, in the ventrodorsal direction. Small eye hooks were attached on the lateral aspects of each vertebra (C4-5, C5-6, C6-7) to guide the preload cable in compression.

### Biomechanical testing procedure

Each vertebral column construct was loaded into a custom-designed testing apparatus at the Engineering Center for Orthopedic Research Excellence (ECORE), University of Toledo, OH. A Plexiglas fixture was attached to the C3 vertebra using a long threaded rod of 4 mm in diameter through the already drilled hole in the ventrodorsal direction and secured with nuts. The rod was used to load the vertebral column in flexion and extension by applying pure moments. The moment (2 Nm) was created by applying weights at a 10-inch distance from the center of the C3 vertebra in the ventral and dorsal aspects of the vertebral column.

A compressive and tensile load of 20 N was applied to the cervical vertebral columns, in neutral position. The neutral position was defined by the straight position of the spine, without load or movement. In order to determine the dimensions of the intervertebral foramina, oil based clay (EZ Shape, Polyform Products Company) was used to fill all foramina on both sides from C4-5 to C6-7. This mold was taken during all loading modes (neutral with no load, neutral with traction, neutral with compression, extension, flexion) and the testing was performed for all 16 vertebral columns. A total of 2880 measurements were obtained from 480 molds of the 96 intervertebral foramina of the 16 cervical vertebral columns (6 foramina times 16 dogs). Each mold was color-coded indicating a specific anatomic location. The molds were then stored in plastic boxes and frozen at −20 °C. The vertical and horizontal dimensions were then measured from the frozen molds.

### Dimensions of the intervertebral foramina

**Fig. 2:**
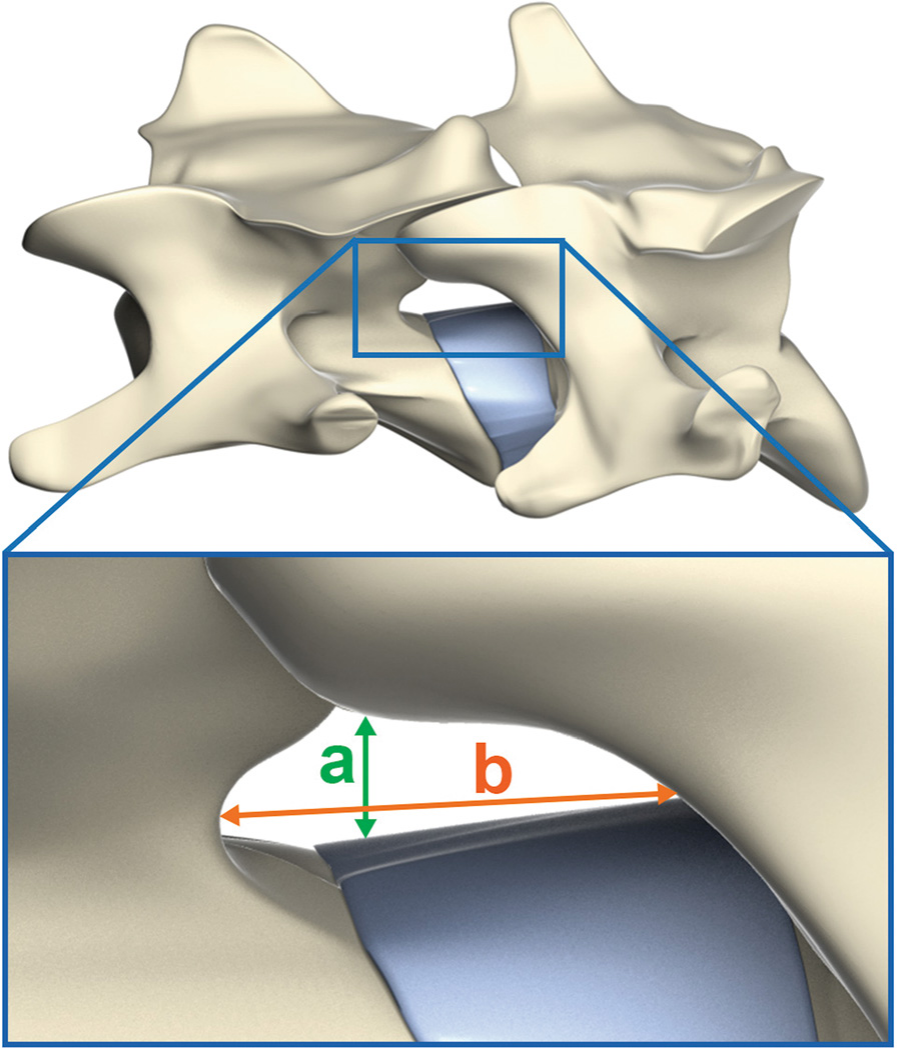
Reference points to measure the dimensions of the cervical vertebral foramina of dogs. **a**, Measurement of foraminal height at C4-5, C5-6 and C6-7. **b**, Measurement of foraminal width at C4-5, C5-6 and C6-7

The vertical and horizontal dimensions of the foramina were measured at each intervertebral level from C4-5 to C6-C7 on both sides. The vertical dimension, or foraminal height, was measured as the highest distance in the ventrodorsal direction. The horizontal dimension, or foraminal width (interpedicular diameter of the vertebral foramina), was measured as the highest distance in the craniocaudal direction (Fig. 2). A digital precision caliper (Digimatic Caliper – Mitutoyo America Corporation), was used to obtain all measurements and the measurements were repeated three times. The caliper accuracy was 0,01 mm.

### Statistical methods

A random-effects linear regression model was used to compare all combinations of movement and load for group 1 and group 2. This analysis was run using 2 statistical models: one for width and the other for height dimension. The regression model included groups (group 1 vs. group 2), movement (neutral, extension, and flexion), load (none, compression, and traction), and side (left vs. right). There was no interaction between groups and movement or between groups and load. Thus, the results shown are independent of the group of the dog meaning that the results are the same whether the dog is group 1 or group 2. The *P*-values were adjusted using the Holm’s procedure to conserve the overall type I error at 0.05 due to the multiple comparisons.

In order to evaluate the reliability of the morphometric evaluation, all measurements were repeated 3 times with at least 1-week interval between measurements. Intraobserver agreement was estimated using the proportion of total variance that is between subjects (rho) using a variance components model based on a random-effects linear regression. If rho is close to 1.0 then the observations are in agreement, whereas if it is close to 0 then there is little agreement. The data were then analyzed using Stata 12.0. (Stata Corporation, College Station, TX).

## Abbreviation

CSM: Cervical spondylomyelopathy
